# Discovery of transgene insertion sites by high throughput sequencing of mate pair libraries

**DOI:** 10.1186/1471-2164-15-367

**Published:** 2014-05-14

**Authors:** Anuj Srivastava, Vivek M Philip, Ian Greenstein, Lucy B Rowe, Mary Barter, Cathleen Lutz, Laura G Reinholdt

**Affiliations:** Computational Sciences, The Jackson Laboratory, Bar Harbor, ME USA; Genetic Resource Sciences, The Jackson Laboratory, Bar Harbor, ME USA; Genome Technologies, The Jackson Laboratory, Bar Harbor, ME USA

**Keywords:** High-throughput sequencing, Mate pair library, Transgenic, Transgene insertion sites

## Abstract

**Background:**

Transgenesis by random integration of a transgene into the genome of a zygote has become a reliable and powerful method for the creation of new mouse strains that express exogenous genes, including human disease genes, tissue specific reporter genes or genes that allow for tissue specific recombination. Nearly 6,500 transgenic alleles have been created by random integration in embryos over the last 30 years, but for the vast majority of these strains, the transgene insertion sites remain uncharacterized.

**Results:**

To obtain a complete understanding of how insertion sites might contribute to phenotypic outcomes, to more cost effectively manage transgenic strains, and to fully understand mechanisms of instability in transgene expression, we’ve developed methodology and a scoring scheme for transgene insertion site discovery using high throughput sequencing data.

**Conclusions:**

Similar to other molecular approaches to transgene insertion site discovery, high-throughput sequencing of standard paired-end libraries is hindered by low signal to noise ratios. This problem is exacerbated when the transgene consists of sequences that are also present in the host genome. We’ve found that high throughput sequencing data from mate-pair libraries are more informative when compared to data from standard paired end libraries. We also show examples of the genomic regions that harbor transgenes, which have in common a preponderance of repetitive sequences.

**Electronic supplementary material:**

The online version of this article (doi:10.1186/1471-2164-15-367) contains supplementary material, which is available to authorized users.

## Background

Transgenic animals are fundamental tools for basic biological research and are widely utilized in the biotechnology and agricultural industries [1]. Since the first transgenic mice were produced by microinjection of DNA into single cell embryos, *i.e.* zygotes, in 1981 [2], transgenesis has become a reliable and powerful method for the creation of new research tools. In mice, nearly 6,500 (data from the Mouse Genome Database, [3]) transgenic alleles have been created for a variety of purposes including tissue specific expression of fluorescent proteins or other “reporters”, tissue specific expression of recombinases (*e.g.* cre recombinase, which is widely used for conditional gene ablation) and for expression of human disease genes [4].

The primary method for creating transgenic animals involves microinjection of purified supercoiled or linearized DNA, i.e., transgene, consisting of genomic or cDNA sequence and, in some cases, residual cloning vector sequence [2]. The injected transgene randomly inserts into the zygotic genome, typically as a multiple copy array. For the majority of transgenic animals, expression of the transgene in resulting founder lines is carefully tested and founder lines with favourable expression levels in the desired tissues are then selected. However, the transgene insertion site is not typically characterized because traditional methods for transgene insertion site discovery are either expensive and/or offer low resolution (DNA FISH) or are complicated by the multicopy nature of the inserted sequences (inverse PCR). However, without this information the position of the insertion site with respect to known genes or regulatory regions and any potential phenotypic complications arising from the proximity of the insertion to these functional elements cannot be established. In addition, rearrangement of the transgene or deletions/rearrangements of the host genome at the insertion site also remain undiscovered. When the transgene insertion site is unknown, genotyping assays to distinguish zygosity must rely on more expensive quantitative PCR approaches based on copy number [5]. These costs are compounded for research animal repositories like The Jackson Laboratory, which manages over 3500 unique strains of transgenic mice.

There is also evidence that transgene expression is impacted by chromosomal location. Chromosomal regions that are subject to epigenetic modification can have a direct impact on transgene expression from one generation to the next. Variability in transgene expression between generations, sexes, environments, or genetic backgrounds is not unusual [6] and is related to copy number where higher copy number is associated with high expression (e.g. [7]). However, depending on the insertion site, higher copy number can also result in epigenetic modification and transgene silencing [8]. Thus, to fully understand how insertion site might contribute to phenotypic outcomes, to more cost effectively manage transgenic strains and to fully understand instability in transgene expression, routine identification of transgene insertion sites is desirable.

Recently, successful transgene insertion site discovery using paired end high throughput sequencing has been reported [9, 10]. Data analysis takes advantage of split reads and discordant mapping of paired reads that are characteristic of reads mapping to insertion sites. However, as with earlier laboratory based approaches (e.g. inverse PCR), the multi-copy arrays that are characteristic of many insertion sites contribute to a low signal to noise ratio in the resulting data, which in turn necessitates high coverage. Additionally, small fragment libraries (typically <400 bp inserts) that are commonly used for paired end sequencing result in a low representation of mapped read pairs that successfully span either end of the transgene insertion. While data on the nature of transgene insertion sites are still emerging, our experience as well as published data from other laboratories show that many transgenes insert in regions that are rich in repetitive sequences like LINE and SINE elements, which in turn negatively impacts the percentage of uniquely mapping reads around the insertion site [9, 11]. Enrichment for sequences in and around a chromosomal rearrangement, whether it’s a translocation breakpoint or a transgene insertion, prior to paired end sequencing is an effective strategy for solving the signal to noise problem by effectively increasing coverage around the insertion or rearrangement [9, 12]. However, this approach requires the manufacturing of a custom array or probe pool for each unique strain, which becomes cost prohibitive for large sets of unique strains.

Here, we describe a methodology for transgene insertion site discovery using high throughput sequencing of libraries made from mate pair sequences that span larger genomic distances [13]. Mate-pair library sequencing is similar to paired end sequencing in that DNA fragments can be sequenced from both ends. However, mate-pair approaches have the added advantage of providing paired sequence from the ends of comparatively large DNA fragment sizes, which promotes the recovery of reads that span insertion sites. We show that 3–5 kb fragment size mate-pair libraries and a scoring scheme specifically designed for transgene insertion site discovery allowed us to successfully discover transgene insertion sites in two widely used mouse models of Amyotrophic Lateral Sclerosis (ALS). We have found that identification of integration sites from mate-pair data has high signal to noise ratio when directly compared to similar analysis on typically short fragment paired end libraries.

## Results and discussion

Illumina high throughput sequencing was done on paired end (~18X theoretical coverage, see Table 1) and mate pair libraries (~32X theoretical coverage, see Table 1) made from genomic DNAs from two widely used transgenic mouse models of Amyotrophic Lateral Sclerosis (ALS), SOD1-G93A and Prp-TDP43^A315T^ [Table 1]. The transgene sequence for SOD1-G93A includes a 14.5 kb region from the human SOD1 locus (chr21:33026936–33043105; GRCh37, hg19) and is present in transgenic animals at relatively high copy number (absolute copy number is unknown) [14, 15]. The transgene sequence for Prp-TDP43^A315T^ contains the human full length A315T mutant TAR DNA binding protein (TARDBP or TDP-43, NM_007375, p.Ala315Thr) cDNA inserted into the XhoI cloning site of the cloning vector MoPrp.Xho (ATCC#JHU-2, which includes the mouse prion protein, Prp, expression cassette) and the copy number is unknown [16].

**Table 1:** **Coverage estimates and orphaned reads mapping statistics**

Categories	SOD1-G93A (PE)*	Prp-TDP43^A315T^(PE)	SOD1-G93A (MP)*	Prp-TDP43^A315T^(MP)
Total number of reads	478630820	474440050	900015938	822321006
Quality filtered reads	370691294	369292346	675672452	662089438
Raw coverage^#^	17.73	17.57	33.33	30.46
Analysis coverage^#^	13.73	13.68	25.02	24.52
Orphaned reads mapped to *Mus*	388	2371	8034	8386
Reads found in the candidates insertion site region	3	5	113	99

*PE: Paired end library.

*MP: Mate-pair library.

^#^Raw coverage corresponds to the sequencing reads generated from machine. Analysis coverage is calculated from quality filtered reads and this dataset is used for insertion site discovery.

### Paired-end sequencing

Paired end data were mapped to the transgene sequences, and read pairs with only one end mapping to the transgene sequence were identified based on SAM flags. The unmapped mates (orphaned reads) of these pairs were then mapped to the mouse genome (MGSCv37, mm9).

Previously published DNA FISH data showed the SOD1 transgene insertion site to be on Chromosome 12 (MMU12) and our DNA FISH data for Prp-TDP43^A315T^ (Additional file 1) showed the TDP43 insertion site to be on Chromosome 9 (MMU9) [17]. Using these results as a guide, all reads mapping to the relevant chromosome were extracted using a Perl script and candidate insertion sites were nominated based on the presence of overlapping reads with mates mapping to the transgene sequence. The paired end data were sufficient, in combination with the DNA FISH data, to nominate an insertion site on MMU12 for the SOD1-G93A transgenic. However, only 3 out of 478,630,820 100 bp reads were informative, indicating an insertion site at Chr12:97,165,800 (MGSCv37, mm9) (Table 1, Additional file 2) which was subsequently validated by PCR and Sanger sequencing.

For Prp-TDP43^A315T^, we were unable to nominate a candidate insertion site from the paired end library data on MMU9. For both SOD1-G93A and Prp-TDP43^A315T^, the paucity of informative reads (0.7 and 0.2%, respectively of orphaned reads) was due to a combination of factors including the genotype of the samples (hemizygous), the amount of sequencing data generated (~18X theoretical coverage) and the small insert size of the library (~210 bp).

Moreover, in the case of Prp-TDP43^A315T^, the transgene consists of a mouse expression cassette (*Prnp*) harbouring a human mutant TDP43 cDNA. Therefore, informative reads consisted of reads representing the transgene as well as reads representing the endogenous mouse *Prnp* locus (Additional file 3). Indeed, when the data were mapped to the mouse genome, a large cluster of reads (read depth > 200) was found at the endogenous *Prnp* locus on MMU2. Conversely, the SOD1 transgene consisted of the human SOD1 locus, such that all reads consisting of human SOD1 sequence were informative.

### Mate pair library sequencing

To increase the signal to noise ratio, mate pair libraries from ~3.8-3.9 kb fragments were constructed and sequencing data were generated at ~32X theoretical coverage. The mate pair library data were analysed using an approach similar to that described above for the standard small fragment paired end libraries, where the data were first mapped to the transgene sequence and then orphaned reads were mapped to the mouse genome (MGSCv37, mm9). For both transgenic strains, nearly 8000 orphaned reads were mapped to the mouse genome, of which ~1.3% were found to be in candidate region (Additional file 4 and Additional file 5).

### Insertion site scoring procedure

To identify the most significant clusters of aligned reads a transgene insertion site scoring procedure was created (see material and methods). This scoring procedure takes a tab delimited text file with 13 fields. A screen-shot of a snippet of a file is shown in supplementary material (Additional file 6). The transgene insertion scoring procedure first divides the genome into blocks and determines the number of reads mapping in each block. The genome-wide threshold for significance for transgene insertion site scores is calculated by random alignments to the reference mouse genome (see Materials and Methods).

**Figure 1:**
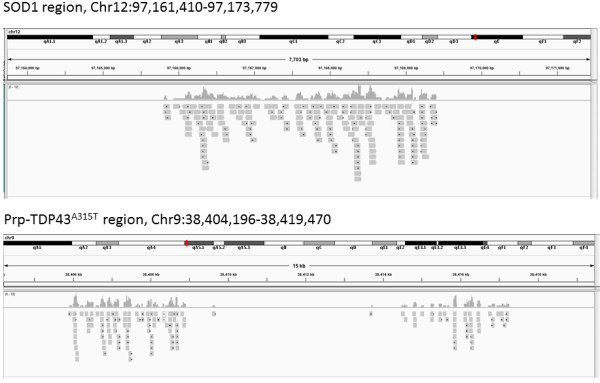
**IGV (Integrated Genome Viewer) view of mate pair library data aligned to the mouse (MGSC37, mm9) genome.** Significant clusters of reads map on MMU12 (SOD1-G93A) and MMU9 (Prp-TDP43^A315T^) each span ~3.8-4 kb, as expected based on the insert size of the mate pair library.

**Figure 2:**
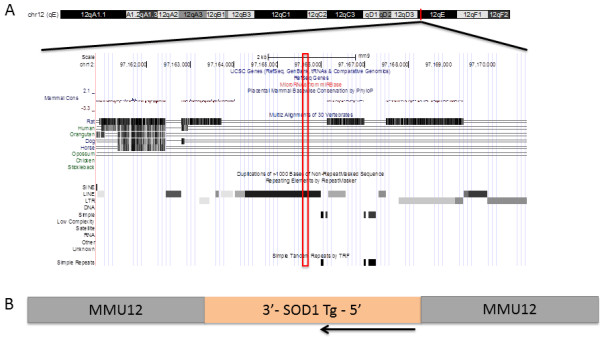
**The SOD1-G93A insertion site on MMU12.** The SOD1-G93A transgene insertion site is at Chr12:97,165,800. In this region, there are no annotated coding sequences, nor is there functional non-coding sequence, as evidenced by the lack of evolutionarily conserved sequence. Instead, this region contains a number of low complexity LINE elements and simple repeats **(A)**. Direct sequencing of the insertion site revealed the orientation of the last copy of the transgene in the multi-copy array with respect to the chromosome **(B)**. Orientation of the chromosome is depicted proximal to distal (left to right) in both panels.

**Figure 3:**
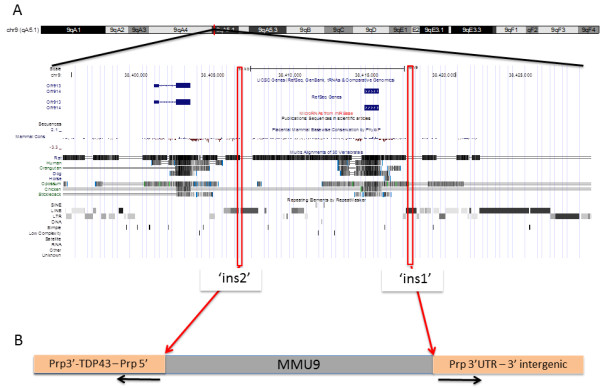
**The Prp-TDP43** ^**A315T**^ **insertion site on MMU9.** There are two closely linked transgene insertion sites on MMU9. These are Chr9:38,417,354 (insertion site 2, ‘ins2’) and Chr9:38,405,898 (insertion site 1, ‘ins1’) (MGSCv37, mm9), respectively. Similar to the insertion site for SOD1-G93A, these insertion sites are not within coding sequences, but are instead embedded in low complexity LINE elements. Moreover, these insertions are in close proximity to olfactory receptor genes, which share a high degree of sequence similarity and are known to evolve by tandem duplication **(A)**. The orientation of the last copy of the transgene in the proximally inserted array and the truncated first copy of the transgene in the distally inserted array are shown in **(B)**. Orientation of the chromosome is depicted proximal to distal (left to right) in both panels.

This analysis process nominated insertion sites for both SOD1-G93A and Prp-TDP43^A315T^ (Figure 1a and 1b). PCR primers designed to flank the proximal and distal ends of each insertion were designed (Additional file 7) and, as expected, these primers amplified products that were unique to animals hemizygous for the relevant transgene (Additional file 8). Capillary sequencing of the PCR products confirmed the insertion sites for SOD1-G93A and Prp-TDP43^A315T^ at Chr12:97,165,800, Chr9:38,417,354 (insertion site 2, ins2) and Chr9:38,405,898 (insertion site 1, ins1) (MGSCv37, mm9), respectively. In both cases, the genomic regions surrounding the transgene insertion sites contain interspersed low complexity repeats, particularly LINE elements (Figure 2 and Figure 3).

### Comparison of paired end and mate-pair data for SOD1-G93A and Prp-TDP43A315T

**Figure 4:**
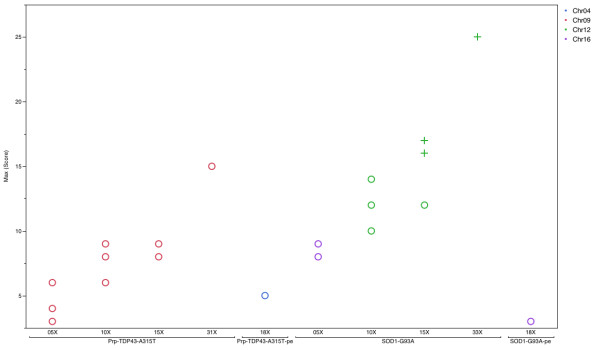
**A summary of coverage analysis.** Top hit in standard paired end and in mate-pair library analysis shown at different coverage (3 technical replicates). The max (score) corresponds to number of reads mapping in 1000 bp blocks. Chr2 is not plotted in the analysis for Prp-TDP43^A315T^ as Chr2 reads represent the endogenous mouse *Prnp* locus. Using the same max (score) the SOD1-G93A and Prp-TDP43^A315T^ transgene has only two points on the plot, at 5X and 15X coverage, respectively.

To demonstrate that mate-pair libraries are more effective than short fragment paired end libraries with comparable sequencing data, we randomly extracted reads from SOD1-G93A and Prp-TDP43^A315T^ mate-pair libraries at 5X, 10X and 15X theoretical genome coverage (3 technical replicates). The entire insertion site identification analysis was performed for data extracted at each of the above coverage levels. A summary of analysis results is shown in Figure 4. In the SOD1-G93A paired end library sequenced at 18X coverage, we could not discern the true insertion site, and a location at MMU16 emerged as the top candidate. However, in the mate-pair SOD1-G93A analysis, the locus on MMU12 (true hit) emerged as top candidate with sequencing data as low as 10X theoretical coverage, and, at 15X coverage, two of the three replicates crossed the significance threshold of 0.1 at the true site (Figure 4). In the Prp-TDP43^A315T^ paired end analysis sequenced at 18X coverage, MMU4 emerged as the top candidate (false hit) but in mate-pair library analysis even at 5X coverage all the top candidate sites belonged to Chr9 (true site). The high scoring, MMU2 hit at the *Prnp* locus was excluded because the transgene contains an expression cassette derived from the mouse *Prnp* gene. From this analysis, it is clear that mate-pair data have a higher signal to noise ratio when compared to paired end data, even at lower sequencing depth. Moreover, where DNA is available from homozygous animals, the depth requirements would, theoretically, be lower by half. While mate-pair library preparation is more than twice the cost of paired end library preparation, mate pair libraries allow for successful insertion site identification at lower coverage (Figure 4). With lower sequencing cost compensating for more costly library preparation, this approach is a cost-effective, reliable solution for routine insertion site identification.

## Conclusions

High throughput sequencing of larger fragment mate-pair libraries at as little as 10X coverage is an effective approach for transgene insertion site discovery when used in combination with an analysis pipeline that provides statistically relevant read cluster identification (Additional file 9). Mate pair libraries provide improved signal to noise ratio when compared to standard fragment paired end sequencing at similar coverage.

The transgene insertion sites for the two transgenic strains used for this pilot project did not contain protein-coding sequences. Instead the transgene insertion sites were associated with LINE (long interspersed elements) elements, which are non-LTR type retrotransposons that are frequently associated with chromosomal rearrangement breakpoints [11]. Transgene insertion sites are frequently associated with chromosomal rearrangements as well as rearrangements and/or fragmentation of the transgene itself [11, 18, 19]. Staged alignment of high throughput sequencing data, where reads are first aligned to the transgene sequence and then only orphaned mates of those reads are aligned to the host genome, simplifies the analysis such that a single cluster of mapped reads indicates a clean insertion, whereas two clusters in close proximity can indicate a deletion in the host genome. More complex rearrangements within the host genome would likely be revealed by various arrangements of clusters in cis and translocations would presumably be revealed by the presence of multiple significant clusters occurring in trans (on different chromosomes) (see also [9]). However, since the approach we used for this study only samples the host genomic sequence immediately flanking the transgene insertion, local inversions of the host genome will be missed.

Direct sequencing of PCR products designed from the mate pair sequences that align within clusters allows for molecular characterization of the transgene ends, which can reveal rearrangements at either end of the transgene array. Additional features of the transgene array itself, including copy number and organization of array subunits are not revealed by using mate pair library sequencing at 10X coverage and would likely require additional sequencing (of standard single end or mate pair libraries) and possibly, de novo assembly.

While only a handful of transgene insertion sites have been molecularly characterized, it is clear that many are associated with LINE and/or SINE elements [9, 11]. While it is possible that these regions may be unusually accessible to transgene integration due to their open chromatin configuration in pronuclear stage embryos [20], we cannot exclude the possibility that this type of integration is enriched in transgenic strains through selective breeding (which would select against insertions that cause lethality or infertility).

## Methods

### Mice

The ALS strains used in this study were SOD1-G93A (The Jackson Laboratory stock #004435) and Prp-TDP43^A315T^ (The Jackson Laboratory, stock #10700). Hemizygous mice from each strain were obtained from The Jackson Laboratory. All procedures involving mice were approved by The Jackson Laboratory’s Institutional Animal Care and Use Committee and performed in accordance with the National Institutes of Health guidelines for the care and use of animals in research. The Jackson Laboratory is accredited by the Association for the Assessment and Accreditation of Laboratory Animal Care (AAALAC).

### DNA FISH and spectral karyotyping

Mitotic spreads were prepared from the bone marrow of Prp-TDP43^A315T^ by removing the bone marrow from femurs into 0.024% colchicine solution (37C for 10 min.). This was followed by hypotonic treatment (0.56% KCl, 15 min.) and fixation of the cell suspension in ice cold 3:1, methanol:glacial acetic acid (2 × 30 min.). Chromosome spreads were prepared by applying the suspension, drop-wise, onto the surface of a clean microscope slide. DNA FISH probes were prepared using nick translation and transgene DNA constructs as template according to the manufacturer’s protocol (Roche Applied Science, #10976776001). Spectral karyotyping (SKY) was also performed according to manufacturer’s protocol (Applied Spectral Imaging, Ltd) and imaging was performed using a Spectral Karyotype system (Applied Spectral Imaging).

### Genomic DNA extraction

Genomic DNA was extracted from hemizygous SOD1-G93A and from hemizygous Prp-TDP43^A315T^ by phenol chloroform extraction of enriched nuclei. Briefly, spleen samples were homogenized in ice-cold Tris lysis buffer (0.02 M Tris, pH 7.5, 0.01 M NaCl, 3 mM MgCl_2_). Homogenates were then incubated in 1% sucrose, 1% NP40 to release nuclei, which were subsequently pelleted by centrifugation at 1,000 rpm, 4°C. Enriched nuclei were then extracted by phenol chloroform in the presence of 1% SDS.

### Paired end library construction

Genomic DNA (1 μg) was fragmented to a peak size of 200 bp by sonicating for 30″ power on, 30″ power off on low power for a total of 10 minutes using a Diagenode Bioruptor UCD-200TM-EX (Denville, NJ, USA). Paired end libraries were constructed using the Illumina (Illumina, San Diego, CA, USA) TruSeq DNA Sample Preparation Kit (part number FC-121-100) with no size selection step. A detailed description of the paired end library prep is available at Illumina website at http://supportres.illumina.com/documents/myillumina/e5af4eb5-6742-40c8-bcb1-d8b350bcb964/paired-end_sampleprep_guide_1005063_e.pdf.

### Mate pair library construction

Mate pair libraries were made using the Illumina Mate Pair v2 kit (part number PE-930-1003). Genomic DNA (10 μg) was fragmented to a peak size of 3 kb at Covaris (Woburn, MA, USA), followed by size selection using a Pippin Prep (Sage Science, Beverly, MA, USA) to tighten the size range around 4 kb. Subsequent fragmentation of the circularized DNA to a peak size of 300 bp was done with a Diagenode Bioruptor UCD-200TM-EX.

A detailed description of mate-pair sequencing is available at http://supportres.illumina.com/documents/myillumina/0a36163e-5fc0-4ae0-a944-a0ee51aa0eb2/matepair_v2_2-5kb_sampleprep_guide_15008135_a.pdf.

The mate pair library prep process includes the paired end library prep process along with additional steps to select the larger fragments, circularize the molecules, re-fragment, and isolate the junction fragments for sequencing. While high throughput sequencing is a rapidly evolving field, using methods that are current at the time of this publication, materials costs for mate pair library prep are about five fold higher than standard paired end library prep. For modest sample numbers, the labor time is about 10 hours for standard paired end library prep and about 16 hours for mate pair library prep. However, the sequencing costs are lower for mate pair because each read pair assays a larger genomic region, meaning that fewer reads are needed to attain adequate genomic coverage for the analysis.

### Illumina sequencing

The sequencing libraries were diluted to 10 nM and used in cluster formation on an Illumina cBot, and PE sequencing was done using Illumina’s HiSeq2000. Both cluster formation and PE sequencing were performed using the Illumina-provided protocols.

### Alignment and orphaned mate identification

Transgene sequences were reconstructed *in-silico* based on the cDNA, vector and/or genomic sequences that were used to generate the transgenes *in-vitro*[14, 16]. The transgene sequence for SOD1-G93A was a 14.5 kb sequence from the human SOD1 locus (chr21:33026936–33043105; GRCh37, hg19) [14] and the transgene sequence for Prp-TDP43^A315T^ was the human full length A315T mutant TAR DNA binding protein (TARDBP or TDP-43, NM_007375, p.Ala315Thr) cDNA inserted into the XhoI cloning site of the cloning vector MoPrp.Xho (ATCC#JHU-2, which includes the mouse prion protein, Prp, expression cassette) [16].

During data analysis (paired end and mate-paired) all samples were subjected to quality control check by *NGSQCtoolkit v 2.3*[21] and samples with base qualities greater ≥ 30 over 70 nucleotides (100 BP reads) were used in the analysis. Quality control reads were mapped to transgene sequences using *bowtie2*[22] short read aligner with default parameters for paired end data and set to --rf, −X 6000 for mate-pair data. Orphaned reads (mate of reads whose one end mapped to transgene) from name sorted sequence alignment map (SAM) [23] file were then extracted using custom Perl scripts. Mapping of orphaned reads to the mouse genome (mm9) was performed by bowtie2 with parameters set to local very sensitive alignment. Then, mapping coordinates of orphaned reads along with various SAM fields were extracted and used as an input to insertion site scoring scheme for significant cluster detection. With respect to the computation time, the quality control step took 12 hours 36 minutes and 42 seconds of wallclock time while utilizing 12 cores and consuming 1.9 GB of RAM on a compute node equipped with four AMD Opteron 6136 processors (8 cores at 2.4 GHz each) and 128 GB RAM for 211,478,020 reads. Alignment to transgene took 1 hour 8 minutes and 56 seconds of wallclock time while utilizing 16 cores and consuming 910 MB of RAM on a compute node equipped with four AMD Opteron 6136 processors (8 cores, 2.4 GHz each) and 128 GB RAM for 168,959,445 Reads.

### Transgene insertion site detection and scoring

To identify potential regions of transgene insertion in an unbiased manner, we applied a segmented window procedure referred to as the *transgene insertion site scoring procedure*. Results obtained from the alignment stage are pre-processed prior to application of the transgene insertion site scoring procedure. Using each reads mapping coordinate, we calculate a distance metric, which we call *distance to next (*DTN*) read*. The DTN measure for a given read along with the host and transgene mapping quality scores are used to remove PCR duplicates (all reads with DTN = 0) and poor quality reads (reads with mapping quality less than 20). Following the application of these filters, the transgene insertion site scoring procedure is initiated. In this procedure, the genome is divided in to approximately 300,000 blocks of 1 Kbp in length. Reads mapping strictly within boundaries defined for each block are retained and is used to define the transgene insertion site score (TISS). The above scoring scheme is aimed at identifying blocks with large numbers of mapped read with small DTN among them, i.e. the signature of a transgene insertion site. With respect to computation time script for insertion site scoring procedure took 5 minutes and 4 seconds of wall clock time while utilizing 1 core and consuming 574 MB of RAM on a compute node equipped with four AMD Opteron 6136 processors (8 cores, 2.4 GHz each) and 128 GB RAM for the filtered dataset obtained from initial 15X sequencing coverage dataset.

### Determination of significance thresholds for transgene insertion site detection and scoring

To determine genome-wide significance thresholds for the transgene insertion site scores, 100 random alignment files (of 100,000 reads each, mapping to mm9) were generated from the datasets of coverage 5X, 10X and 15X. These different coverage datasets were made by first extracting all mouse specific reads from SOD1-G93A entire mate-pair library and then extracting reads from mouse specific fastq files by seqtk package (sample utility at different seed values). The DTN measure was computed for each of the 100 random files above, followed by the application of the transgene insertion site scoring procedure. The maximum window score observed in each randomization was retained to obtain the random distribution of score in the event of no transgene. Quantiles at 99%, 95% and 90% were calculated to determine 0.01, 0.05 and 0.10 genome-wide significance thresholds.

### Experimental verification

Transgene insertion sites were verified using PCR and capillary sequencing (i.e. Sanger sequencing). Primer sequences used for validation are in Additional file 7. Genomic DNAs from hemizygous transgenic animals and non-carrier controls were used for PCR amplification. Depending on the product size, either standard or long range PCR was used. For standard PCR, primers spanning the candidate insertion site were added at a concentration of 0.5 μM to Taq polymerase master-mix (5Prime Inc. Gaithersburg, MD, cat# 2200110). PCR cycling conditions were 95°C for 2:30, 95°C for 0:30, 59.4°C for 0:30, 72°C for 2:00 for 40 cycles, followed by a 9:30 final extension at 72°C. PCR products were cloned using the TOPO® TA Cloning® Kit, (Dual Promoter kit, Life Technologies cat# K4610-20). Clones were screened by restriction digest with EcoR1 (New England Biolabs, Ipswich, MA cat# R3101S) and BamH1 (New England Biolabs cat# R3136S), and sequenced by Sanger sequencing using the M13 bacteriophage forward and reverse primers.

For long range PCR, Phusion High-Fidelity DNA Polymerase (New England Biolabs, cat# M0530S) was used. Final primer concentrations were 0.66 μM. PCR cycling conditions were 95°C for 2:30, 95°C for 0:30, 62°C for 0:30, 72°C for 2:00 for 40 cycles, followed by a 10 minute extension at 72°C. PCR products were cloned using the TOPO® TA Cloning® Kit (Dual Promoter kit, Life Technologies cat# K4610-20). Clones were screened by restriction digest with EcoR1 (New England Biolabs, Ipswich, MA cat# R3101S) and BamH1 (New England Biolabs cat# R3136S), and primers for Sanger sequencing were the M13 bacteriophage forward and reverse primers. Cycle sequencing of DNA samples was performed using Applied Biosystems BigDye Terminator ready reaction kit Version 3.1.

## Availability of supporting data

The material described here is available in the form Perl scripts. A detailed document is also attached describing the analysis protocols.

## Electronic supplementary material

Additional file 1: **DNA FISH analysis and spectral karyotyping of mitotic chromosomes from a Prp-TDP43** ^**A315T**^ **hemizygote.** DNA FISH using a probe generated from the Prp-TDP43^A315T^ construct shows a specific hybridization signal (**A**., red arrow, red spot) on MMU9 (**B**., white arrow). (TIFF 226 KB)

Additional file 2: **SOD1-G93A paired end analysis results.** The score corresponds to number of reads mapping in 1000 bp blocks and the dotted line indicates the significance threshold of 0.1. (JPEG 883 KB)

Additional file 3: **Prp-TDP43** ^**A315**^ **paired end analysis results.** The score corresponds to the number of reads mapping in 1000 bp blocks and the dotted line indicates the significance threshold of 0.1. (JPEG 880 KB)

Additional file 4: **SOD1-G93A mate-pair analysis results.** The score corresponds to number of reads mapping in 1000 bp blocks and dotted line indicates the significance threshold of 0.1. (JPEG 476 KB)

Additional file 5: **Prp-TDP43** ^**A315**^ **mate-pair analysis results.** The score corresponds to number of reads mapping in 1000 bp blocks and dotted line indicates the significance threshold of 0.1. (JPEG 446 KB)

Additional file 6: **Screen-shot of the file used as an input for insertion site scoring scheme.** (TIFF 123 KB)

Additional file 7: **Sequences of both long range and standard PCR primers used for validation of transgene insertion sites.** (XLSX 36 KB)

Additional file 8: **PCR amplification of transgene insertion sites.** PCR was used to experimentally validate the candidate insertion sites for Prp-TDP43^A315T^ and SOD1-G93A. Unique PCR products were amplified from transgenic animals and not from littermate controls. (TIFF 164 KB)

Additional file 9: **Analysis pipeline and relevant scripts used in the analysis.** (ZIP 13 MB)
