# Supplementary figures and images for: Discovery of transgene insertion sites by high throughput sequencing of mate pair libraries
Source: BMC Genomics. 2014 May 14;15(1):367. doi: 10.1186/1471-2164-15-367 (PMC4035081; doi:10.1186/1471-2164-15-367)

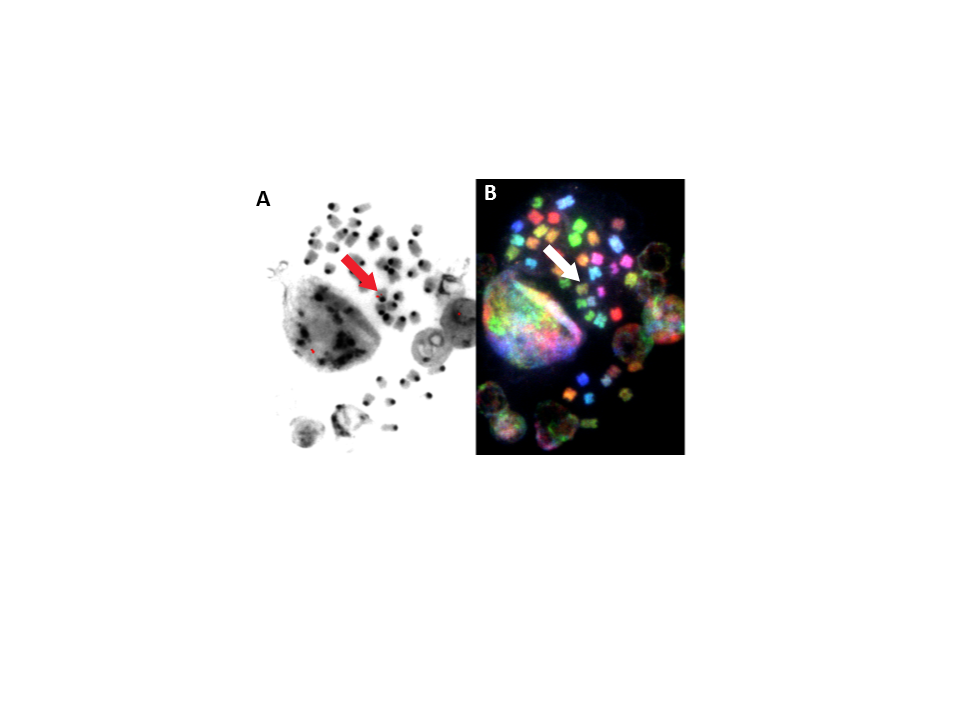

Supplement: Supplementary file 1 — Additional file 1: DNA FISH analysis and spectral karyotyping of mitotic chromosomes from a Prp-TDP43 A315T hemizygote. DNA FISH using a probe generated from the Prp-TDP43A315T construct shows a specific hybridization signal (A., red arrow, red spot) on MMU9 (B., white arrow). (TIFF 226 KB) [file 12864_2013_6050_MOESM1_ESM.tiff]

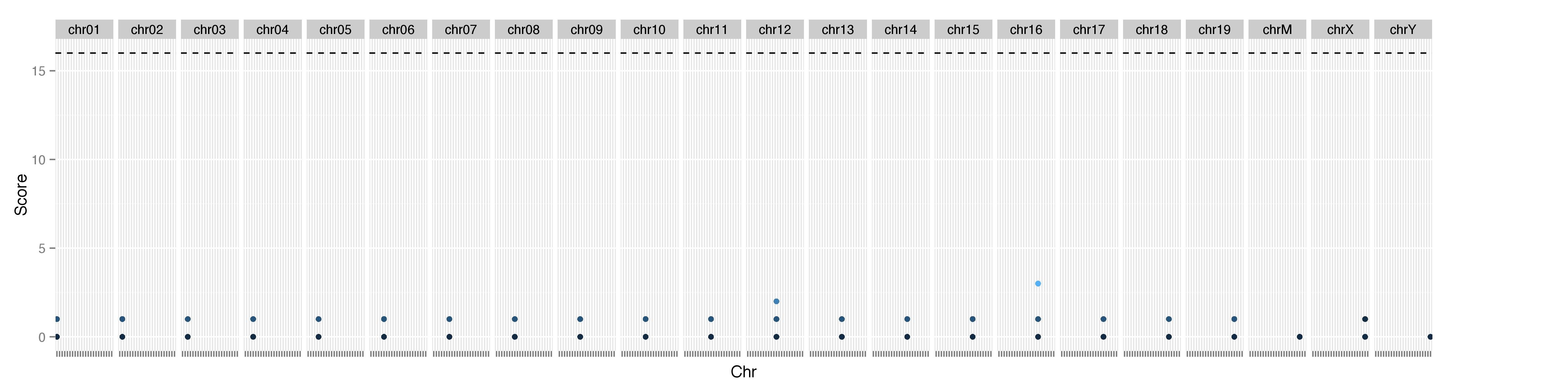

Supplement: Supplementary file 2 — Additional file 2: SOD1-G93A paired end analysis results. The score corresponds to number of reads mapping in 1000 bp blocks and the dotted line indicates the significance threshold of 0.1. (JPEG 883 KB) [file 12864_2013_6050_MOESM2_ESM.jpeg]

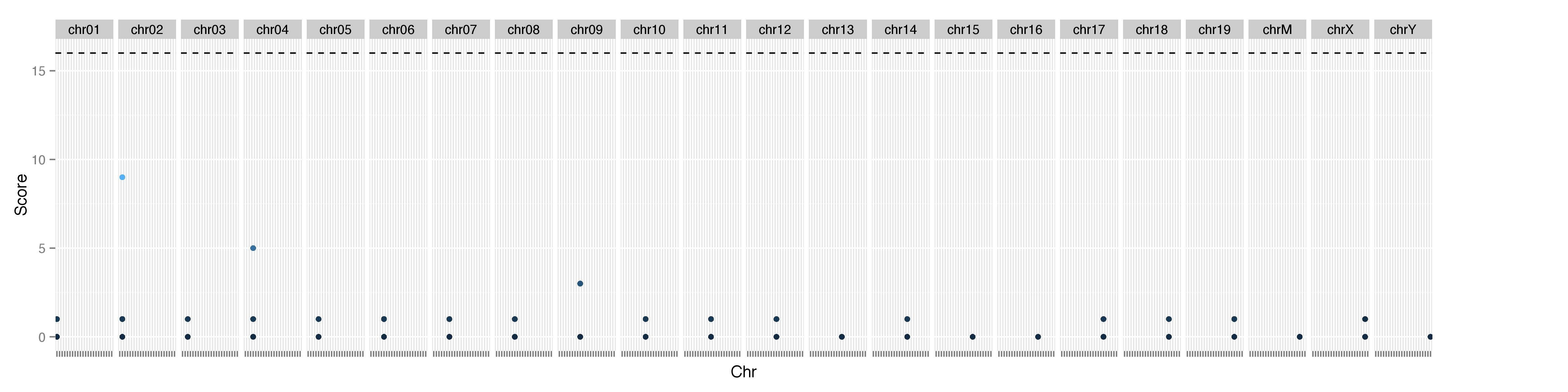

Supplement: Supplementary file 3 — Additional file 3: Prp-TDP43 A315 paired end analysis results. The score corresponds to the number of reads mapping in 1000 bp blocks and the dotted line indicates the significance threshold of 0.1. (JPEG 880 KB) [file 12864_2013_6050_MOESM3_ESM.jpeg]

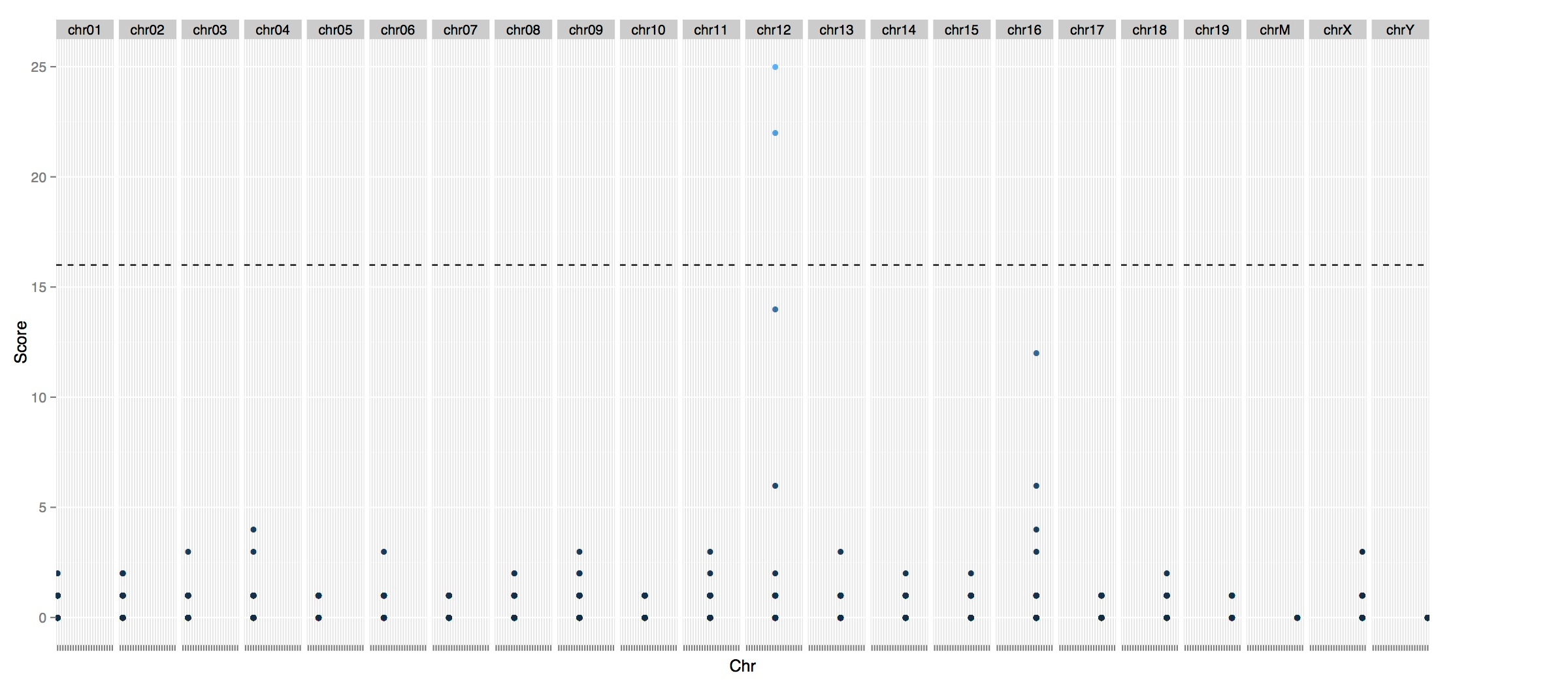

Supplement: Supplementary file 4 — Additional file 4: SOD1-G93A mate-pair analysis results. The score corresponds to number of reads mapping in 1000 bp blocks and dotted line indicates the significance threshold of 0.1. (JPEG 476 KB) [file 12864_2013_6050_MOESM4_ESM.jpeg]

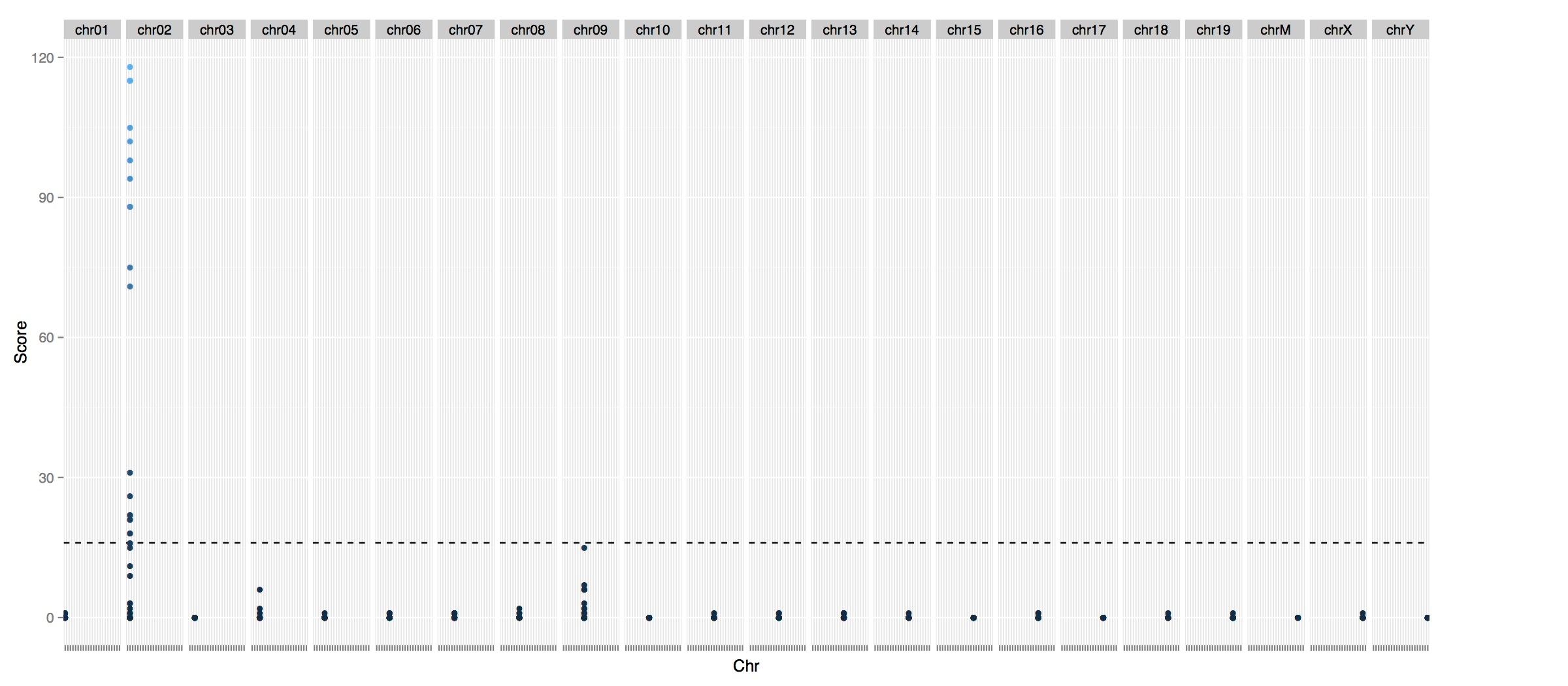

Supplement: Supplementary file 5 — Additional file 5: Prp-TDP43 A315 mate-pair analysis results. The score corresponds to number of reads mapping in 1000 bp blocks and dotted line indicates the significance threshold of 0.1. (JPEG 446 KB) [file 12864_2013_6050_MOESM5_ESM.jpeg]

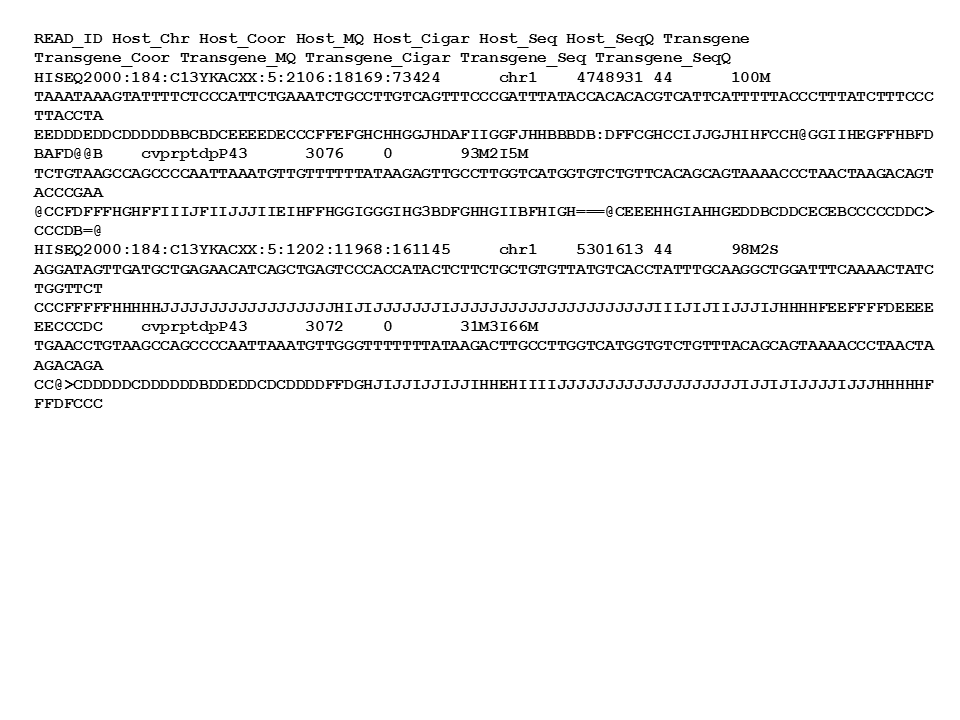

Supplement: Supplementary file 6 — Additional file 6: Screen-shot of the file used as an input for insertion site scoring scheme. (TIFF 123 KB) [file 12864_2013_6050_MOESM6_ESM.tiff]

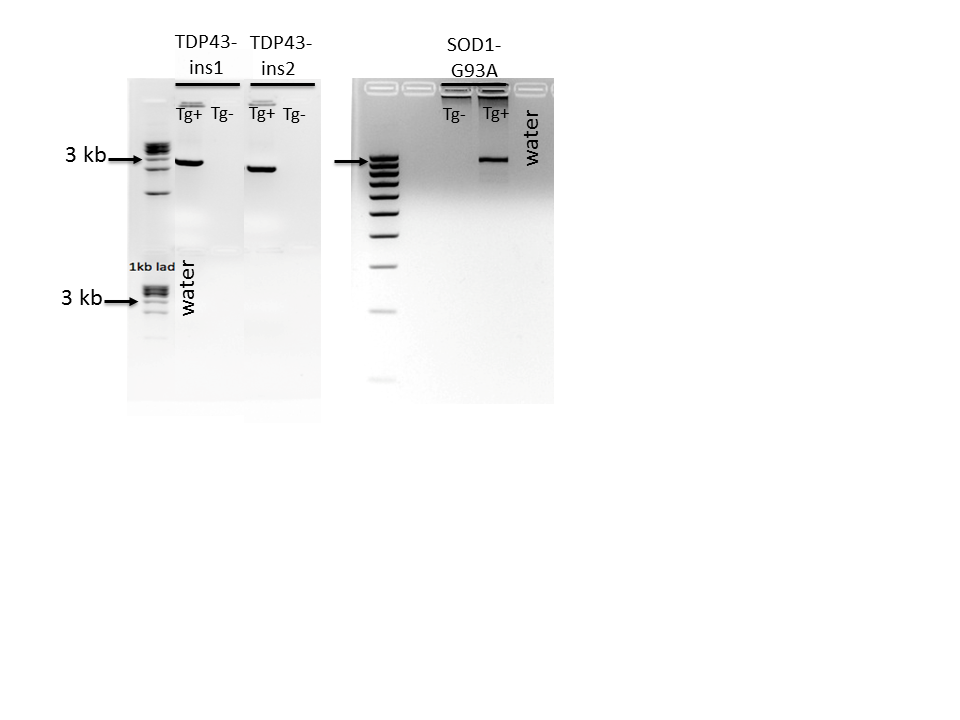

Supplement: Supplementary file 8 — Additional file 8: PCR amplification of transgene insertion sites. PCR was used to experimentally validate the candidate insertion sites for Prp-TDP43A315T and SOD1-G93A. Unique PCR products were amplified from transgenic animals and not from littermate controls. (TIFF 164 KB) [file 12864_2013_6050_MOESM8_ESM.tiff]
