# Supplementary material for: Discovery of transgene insertion sites by high throughput sequencing of mate pair libraries
Source: BMC Genomics. 2014 May 14;15(1):367. doi: 10.1186/1471-2164-15-367 (PMC4035081; doi:10.1186/1471-2164-15-367)
Supplement: Supplementary file 9 — Additional file 9: Analysis pipeline and relevant scripts used in the analysis. (ZIP 13 MB) [file 12864_2013_6050_MOESM9_ESM.zip › Additional_File9/Insertion_Site.docx]

**Transgene Insertion Site Finding Using Mate-Pair Libraries**

**Anuj Srivastava**

**Step1: NGS QC**

**Tool Requirement:**

NGSQCToolkit/2.3

- 1. **Quality Statistics generation and bad read filtering**

**Illumina sequencing; CASAVA 1.8+; mate-pair reads;**

**Usage:**

IlluQC_PRLL.pl -pe <read1.fastq> <read2.fastq> <adaptor_file> <5> –s 30 -c 12 -o filter

**Input:**

- Forward paired end file [required]
- Reverse paired end file [required]
- Adaptor_database file or application library [required]
- FASTQ encoding [required]

**Output:**

Folder named “filter” created here which has the following files

- read1.fastq_filtered (here)
- read2.fastq_filtered (here)
- *_unPaired_HQReads
- *_stat
- *.html,
- 6 plots for each fastq file*_avgQual.png, *_baseCompostion.png, *_QualRangePerBase.png, *_ gcDistribution.png, *_ qualDistribution.png, *_ QualRangePerBase.png,
- *_summary.png (summary plot)

**Parameters Description:**

- -pe: Forward reverse paired end file
- Adaptor_file: Should be replaced by known adaptor sequence used in particular type of sequencing
- FASTQ quality value variants: 5 stands for = Illumina (1.8+) (Phred+33, 33 to 7)
- -s: -cutOffQualScore (The cut-off value for PHRED quality score for high-quality filtering)
- -c: Number of CPUs to be used
- -o: Output will be stored in the given folder

**1.2 Quality based trimming of filtered files**

**Usage:**

TrimmingReads.pl –i <filter/read1.fastq_filtered> -irev <filter/read2.fastq_filtered> –q 30

**Input:**

- Forward paired end file with paired end intact after filtering [required]
- Reverse paired end file with paired end intact after filtering [required]

**Output:**

- read1.fastq_filtered_trimmed (here)
- read2.fastq_filtered_trimmed (here)

**Parameters Description:**

- -i <Forward read/sequence file>
- -irev <Reverse read/sequence file of paired-end data>
- -q | -qualCutOff <Integer> (Only for FASTQ files) Cut-off PHRED quality score for trimming reads from right end (3' end)

**Reference:**

Patel et al. (2012) “NGS QC Toolkit: A Toolkit for Quality Control of Next Generation Sequencing Data. *PloS ONE*

# Step2: Indexing transgene

**Tool Requirement:**

bowtie2

**Usage:**

bowtie2-build transgene.fa index_name

**Input:**

- Transgene sequence in fasta format (fasta header should not have spaces)
- Base name append to each output index file

**Output:**

- 6 index file with *.bt2 extension

**Parameters Description:**

- Transgene sequence file to be indexed
- Base_name to append to each output file

# Step 3: Alignment to transgene

**Tool Requirement:**

bowtie2

## Usage:

bowtie2 -x index_name \

-1 <read1.fastq_filtered_trimmed>

-2 <read2.fastq_filtered_trimmed> \

-X 6000 --rf -p 16 -S out.sam

**Input:**

- Trimmed forward and reverse mate paired fastq files [read1.fastq_filtered_trimmed and read2.fastq_filtered_trimmed here]
- Indexed transgene base name and location [index_name here]

**Output:**

- Alignment file in sam format (out.sam here)

**Parameters Description:**

-X: The maximum fragment length for valid paired-end alignments.

--rf: The upstream/downstream mate orientations for a valid paired-end alignment against the forward reference strand. E.g., if --fr is specified and there is a candidate paired-end alignment where mate 1 appears upstream of the reverse complement of mate 2 and the fragment length constraints ([-I](http://bowtie-bio.sourceforge.net/bowtie2/manual.shtml#bowtie2-options-I) and -X) are met, that alignment is valid. Also, if mate 2 appears upstream of the reverse complement of mate 1 and all other constraints are met, that too is valid. --rf likewise requires that an upstream mate1 be reverse-complemented and a downstream mate2 be forward-oriented. --ff requires both an upstream mate 1 and a downstream mate 2 to be forward-oriented. Default: --fr (appropriate for Illumina's Paired-end Sequencing Assay).

-S: File to write SAM alignments [out.sam here]; this file is sorted by read name

-p: Launch NTHREADS parallel search threads (default: 1).

**Step 4: Extracting orphaned reads**

**Tool Requirement:**

extract_unmapped_mates.pl (make sure script is present)

## Usage:

perl **extract_unmapped_mates.pl**  -in out.sam -gs **gene_name_to_search\|gene2**  -c 04:00:00 -m [anuj.srivastava@jax.org](mailto:anuj.srivastava@jax.org) -o test &

**Input:**

Read alignment file to transgene (bowtie2 outputs this file and it is already sorted by name; if your file is not sorted then you need to sort by name) [Out.sam here]

**Output:**

- This file will have the reads mapped to transgene [test.results here]
- This file will have the sequences of those reads whose one end mapped top transgenes [test.fastq here]

### Parameter description:

- -in: input sam alignment file
- -gs: gene_string_to_search (should be identical to header in fasta file);multiple gene could be searched separated by \|
- -c: wall time of job submission
- -m: E_mail id
- -o: output_name will attach to each file (“test” here)

**Step5: Aligning orphaned reads to Host**

**Shell Command:**

bowtie2 –x mm9_bowtie2_index --local \

--very-sensitive-local -U test.fastq -p 12 \

-S test.host.sam

**Sort: test.host.sam file by coordinates using samtools or picard (The final outcome test.host.sorted.sam)**

**Step 6: Extracting orphaned reads host and transgene location and important alignment fields**

**Tool Requirement:**

get_mapped_mate_info.pl (make sure script is present)

**Usage:**

perl get_mapped_mate_info.pl –i1 transgene_mapped_sam_file –i2 host_mapped_sam_file –o outname

**Input:**

- Transgene mapped sam file [test.results here]
- Host mapped sam file [Test.host.sorted.sam here]

**Output:**

- Output file with orphaned mates host and transgene location and alignment fields [Test.InsertionSites.txt]

### Parameter description:

- -i1: transgene mapped sam file
- -i2: host mapped sam file
- -o: output_name will attach to each file (“**test**” here)

**Step7: Scoring Scheme Part 1:**

**Tool: calc_dist.pl**

**Usage:**

perl calc_dist.pl <input file> <output file> <mapping quality threshold> <distance-to-next threshold>

**Input:**

- File with orphaned mates host and transgene location and alignment fields [Test.InsertionSites.txt]
- Outfile name [filtered.txt here]
- Mapping quality threshold
- distance-to-next threshold

**Output:**

- Filtered file for window score calculation [filtered.txt]

### Parameter description:

- mapping quality threshold [ 20 could be reasonable cut-off]
- distance-to-next threshold [1 could be reasonable cut-off]

**Step 8: Scoring Scheme Part 2:**

**Tool: calc_window_score**

**Usage:**

Perl calc_window_score.pl <input file 1> <input file 2> <output file>

**Input:**

- Filtered file for window score calculation [filtered.txt here]
- Window definition file [for mouse provided with package]

**Output:**

- Outfile with score for each 1000 BP chromosome window

### Parameter description:

- Window definition file
